# Adapting the Facilitating Conditions Questionnaire (FCQ) for Bilingual Filipino Adolescents: Validating English and Filipino Versions

**DOI:** 10.1007/s12187-012-9167-1

**Published:** 2012-10-03

**Authors:** Fraide A. Ganotice, Allan B. I. Bernardo, Ronnel B. King

**Affiliations:** 1 Palawan State University, Puerto Princesa, Philippines; 2 University of Macau, Macau, People’s Republic of China; 3 The University of Hong Kong, Hong Kong SAR, China; 4 Room 101 HOC Science Bldg., Faculty of Education, The University of Hong Kong, Pokfulam, Hong Kong

**Keywords:** Facilitating conditions questionnaire, Validation, Translation, Confirmatory factor analysis, Bilingual, Adolescents

## Abstract

This study examined the applicability of the English and Filipino versions of the Facilitating Conditions Questionnaire (FCQ) among Filipino high school students. The FCQ measures the external forces in students’ social environments that can influence their motivation for school. It is composed of 11 factors: university intention, school valuing, parent support, teacher support, peer help, leave school, pride from others, negative parent influence, affect to school, negative peer influence, and positive peer influence. It was translated into conversational Filipino. Seven hundred sixty-five high school students answered one of the two language versions. Both within-network and between-network approaches to construct validation were used. Confirmatory factor analyses (CFA) of the two versions showed good fit. Results of the multigroup CFA indicated that there was invariance in terms of factor loadings for the two versions. Results of the between-network test also showed that the factors in the FCQ correlated systematically with theoretically relevant constructs. Taken together, this study supports the applicability of the FCQ for use with Filipino bilingual adolescents.

## Introduction

Participation in basic education is an important social development indicator in many countries. In the Philippines, basic education is considered a basic human right, and as such the Constitution mandates the government to provide free basic education (primary and secondary) for all. But data from the country’s Department of Education (Albert et al. 2012) indicate that the participation rate is only 88.1 % in primary education and 60.7 % in secondary education. A survey of the National Statistics Office (2008) indicates that “lack of personal interest” in schooling was the reason given by 44.59 % of out-of-school high-school age children, which underscores the importance of student interest and motivation in keeping Filipino students in school. This observation is likely to be true in many other countries, and this is why understanding the factors that facilitate academic motivation among students is one of the valued goals of educators (Grolnick et al. 2007). In particular, attention has focused on understanding the factors that contribute to the lack of academic motivation among adolescents in high school (Brown-Wright et al. 2011; Legault et al. 2006).

Numerous studies have been conducted with the intent of finding better ways of motivating learners, and these studies often involve measures of student motivation. Most of these studies have focused on the assessment and examination of internally-referenced motivational constructs such as goals (Elliot 2005; Elliot and McGregor 2001; Pintrich 2000), self-efficacy (Bandura 1997, 2011), self-concept (Marsh and Craven 2008), emotions in school (Pekrun et al. 2002), and value for schooling (Bernardo 2003; Maehr and McInerney 2004), among others. However, despite the importance of these internal forces, externally-referenced forces also facilitate or inhibit motivation in school (Benner and Mistry 2007; Deci et al. 1991). For example, research has indicated that significant others can influence academic motivation. Beliefs held by parents (Aunola et al. 2003; Hill and Craft 2003), the quality of interaction with peers (Wentzel et al. 2004), and the nature of feedback and support from teachers (Reeve 2006; Reeve and Jang 2006) influence students’ motivations in school. Thus, in order to understand why students may or may not be motivated in school, it is important to know how students perceive these internally and externally-referenced factors that shape their school motivation.

The Facilitating Conditions Questionnaire (FCQ, McInerney et al. 2005) is one of the few paper-and-pencil tests that seek to assess how students perceive these different external forces that may shape their motivation in schools together with some internally referenced factors. This study looks into the validity of the English and Filipino versions of the FCQ for use among bilingual Filipino students. Nearly all Filipino students are at least bilingual, and most are actually multilingual. Recent surveys (e.g., Grimes 2002) indicate that most Filipinos speak at least one Philippine language or dialect. Moreover, the Philippines adopted a bilingual education policy in 1974 (Bernardo 2004); and this policy mandates the use of Filipino and English in instruction at all levels. As a result, most Filipino students have adopted Filipino and English as languages of discourse in most forms of social interaction (Gonzalez et al. 2000), and especially in formal education. A recent survey (Social Weather Stations 2008) indicates that among Filipinos with some high school education, 74 % can understand spoken English, 73 % can read English, 52 % can write in English, and 32 % can speak in English. In this regard, it is important to study the two language versions of the FCQ to see whether these adequately assess the same factors that facilitate student motivation among the bilingual Filipino learners.

### The Facilitating Conditions Questionnaire (FCQ)

The FCQ is a 55-item instrument designed to measure 11 factors. It is anchored on a 5-point Likert scale from 1 (*strongly disagree*) to 5 (*strongly agree*). The subscales of the FCQ and sample items include:

- *University intention*: degree to which important others think that one should go to university or not (“I intend to go to college or university”)
- *School valuing*: the degree to which one values school for future outcomes (“Education is important to me to get a job”)
- *Parent support*: the degree to which one perceives their parents to be supportive of their academic tasks (“My mother helps me with my school work”)
- *Teacher support*: the degree to which one perceives their teachers to be supportive of their studies (“Teachers are positive to me at school”)
- *Peer help*: the degree to which one receives help from their friends in their school work (“Some of my friends help me with my school work”)
- *Leave school*: the degree to which significant others such as parents think that one should leave school (“My mother doesn’t mind if I leave school when I want to”)
- *Pride from* *others*: degree to which significant others feel proud of one’s schoolwork (“It’s important for my father to be proud of my school work”)
- *Negative parent* *influence*: degree to which parents exert a negative impact on one’s schooling (“My father doesn’t pay any attention when I bring home report cards”)
- *Affect to* *school*: degree to which one likes school (“I like studying”)
- *Negative peer* *influence*: degree to which one’s friends are disengaged from school (“Some of my friends tell me I should leave school when I can”), and
- *Positive peer* *influence*: degree to which one’s friends are engaged in academics (“Most students in my class will go on to college or university”).

The FCQ has been used in previous studies to examine how perceptions of facilitating and inhibiting conditions may differ between elementary and secondary students (see McInerney et al. 2005) and to compare facilitating conditions profiles among cultures (e.g., McInerney 2008) among others. However, most of these previous studies have been conducted among Western students (McInerney et al. 2005). It has also been used among minority cultural groups such as Lebanese and Australian aboriginal students. However, the cross-cultural applicability of this instrument was not strictly examined as those studies only focused on Cronbach’s alpha reliabilities, which seem to constitute a weak basis for assuming that the FCQ is valid in cross-cultural settings.

### Cross-Cultural Issues

There is still a dearth of studies with regard to the psychometric properties of this instrument in the Asian setting. As Maneesriwongul and Dixon (2004, p. 175) noted, “Research instruments must be reliable and valid in each culture studied.” An inherent danger in using instruments in other cultural settings is the assumption made by researchers that items and constructs developed and standardized on one particular cultural group are broadly universal when in fact there is no attempt made to demonstrate the applicability of the constructs or instruments used to new groups (see McInerney et al. 2001). There are a variety of methodological and conceptual difficulties involved in capturing behaviors, attitudes and values in cross-cultural studies (van de Vijver and Hambleton 1996; van de Vijver and Poortinga 1982). Thus, it is important that the issue of validity is addressed before the results of such psychological tests with different cultures can be interpreted (Hambleton 2001; van de Vijver and Tanzer 2004). The cross-cultural applicability of the FCQ still needs further exploration especially when administered to Asian students. Therefore, in this study, we explored the cross-cultural validity of the English and Filipino translations of the FCQ among adolescent Filipino students in high school. As mentioned earlier, most Filipino students are multilingual, however, we limit our investigation to the Filipino and English versions of the FCQ, as these are the two languages that are most extensively used in the Philippine school environments. Moreover, although the FCQ is developed to be used for children in upper primary and secondary education, we also limit the current investigation to the validity of the FCQ with high school students.

### Approaches to Construct Validation

The present study adopts a construct validation approach (Marsh 1997) to the empirical assessment of the structure of the Filipino and English versions of the FCQ. Studies that adopt this approach can be classified as either within-network or between-network studies. Within-network construct validation, also called internal construct validation refers to the examination of the factor structure and factor correlation matrix. On the other hand, between-network or external construct validation approach entails examining patterns of relationships between the scales and other theoretically related constructs (Marsh 1997). Combining the within- and between-network approaches is considered more robust compared to other procedures which only use one of the two. This allows researchers to examine the factor structure of the questionnaire while at the same time examining its relationship with other externally relevant measures making the validation procedure more robust. The present study uses both approaches. First, we conduct a within-network study using confirmatory factor analysis to test the 11-factor structure of the FCQ separately in the two language versions followed by multigroup confirmatory factor analysis to assess the invariance of the instrument across the two versions. Consistent with the construct validation approach, it is not only important to address validity within an instrument (within-network validity) but it is also imperative to explore the possible differential relationships between the 11 factors and a set of theoretically relevant measures (between-network validity). In our study, we assessed how the factors in the FCQ are related to other theoretically-relevant variables such as the sense of self. We assumed that the positive dimensions of the FCQ would be positively related to the positive sense of self dimensions.

## The Present Study

The aim of the present study was to assess the applicability of the English and Filipino versions of the FCQ among bilingual Filipino adolescents in schools. Both within-network and between-network approaches to construct validation were adopted. For the within-network test, we conducted a confirmatory factor analysis to determine the fit of the data. We also employed multigroup confirmatory factor analysis to assess the invariance of the FCQ across the two language versions. For the between-network test, we assessed the correlation of the factors in the FCQ with Sense of Self Scale (SOS; Ganotice and Bernardo 2010; King et al. 2012a, b) which includes sense of purpose, positive self-concept, and negative self-concept. We hypothesized that the positive dimensions of the FCQ would be positively correlated to the sense of purpose and positive self-concept scales.

## Method

### Participants

The participants of this study were 765 high school students from five public and private learning institutions (two in the National Capital Region, one from Region IV-A and two from Region IV-B). The students were distributed from first year to fourth year high school and all the students present during data-gathering day were involved in answering the questionnaires.

Specifically, there were 378 participants (160 males and 218 females) who completed the English version, and 387 participants (141 male and 246 female) who completed the Filipino version. Participants were randomly assigned to one of the two groups. The average age of those completed the English version was 14.62 years (*SD* = 1.39 years) and the median age was 15 years, whereas the average age of those who answered the Filipino version was 13.98 years (*SD* = 1.41 years) and the median age was 14 years. All the participants were required to complete the inventory as part of the class requirement. Convenience sampling was used to recruit the participants in this study.

### Measures

This study used the original 55 items of the Facilitating Conditions Questionnaire with 11 factors (See Appendix for the English and Filipino versions). All the items in the questionnaire are anchored on a 5-point Likert-type scale where respondents just selected/marked their response ranging from 1 (strongly disagree) to 5 (strongly agree). The items were arranged in cyclic order where all the first items of the eleven factors comprised the items 1-11, and all the second items of the eleven factors were positioned to numbers 12–22. The same procedure is done to the rest of the items. This is especially designed to prevent potential “set effects” in which students identify contiguous items as items measuring similar attributes (Bong 1997).

The Sense of Self Scale (King et al. 2012a, b) which measures sense of purpose for schooling, positive self-concept, and negative self-concept was also administered to test for between-network validity.

*Translation procedure*. Forward and back translations were carried out in order to develop the Filipino version of the said instrument. A committee approach to translation was used in this study where the translation team composed of four members did the translation from the original English into Filipino. The members were all enrolled in a PhD in Educational Psychology program and had different fields of specialization in their Master’s degrees (counseling psychology, early childhood education, school administration and clinical psychology). One member had experience in instrument translation and had worked as an English teacher for foreign students. An external auditor observed the dynamics of the translation and provided feedback as to how to further improve the group dynamics involved in the process.

The original English version of the instrument was provided early on to the four translators. They were requested to translate the items (forward translation) individually in Filipino. When the group met with individual translations, they were asked to reach a consensus on each of the items after hearing from all the members.

In keeping with the idea that none of the translation techniques is perfect, back-translation was the next technique performed in this study. Back-translation, a highly recommended technique by experts in cross-cultural research (Champman and Carter 1979; Maneesriwongul and Dixon 2004), is where the target language version is translated back into the source language version in order to verify translation of the research instrument. For the back-translation, each committee member was given the chance to give his/her translation and other members also commented on the items until the group reached a consensus. Some of the Filipino translations were adjusted or revised so they more closely reflect the original English items. Some of the Filipino translations contained words from the original English form. These English words were retained because these were more commonly used by high school students in their casual conversations compared to the corresponding Filipino translations. In such cases, the Filipino translations were code-mixed statements with English lexical units used within sentences in Filipino syntax.

*Statistical analysis*. For the within-network study, confirmatory factor analysis (CFA) was used. CFAs assess the extent to which the observed indicators (items) reflect the structure of the underlying constructs. CFAs allow the researcher to specify not only how many factors are measured by a given set of items but, also, which items function as indicators of which factors.

As discussed earlier, the FCQ is composed of 55 items with 3 to12 indicators for each factor (please refer to Table 1). Bagozzi and Heatherton (1994) concluded that when there are more than four or five indicators per factor in a large sample, it is quite likely to lead to an unsatisfactory fit in the measurement model. To address this issue, we aggregated the items to form item “parcels” as indicators in the CFA. Parcels were formed by randomly combining two to four items in each scale. For the first factor of the FCQ composed of 5 items, the first item parcel was comprised by the following items: 1, 3, and 5. The second item parcel includes items 2, and 4. There were 22 parcels which were subsumed by 11 factors: 2 parcels from 5 items of the first factor; 3 parcels from 9 items of the second factor; 2 parcels from 6 items of the third factor; 2 parcels from 6 items of the fourth factor, 2 parcels from 5 items of the fifth factor, 2 parcels from 4 items of sixth factor, 2 parcels from 4 items of the seventh factor, 2 parcels from 4 items of the eight factor, 1 parcel from 3 items of the ninth factor, 2 parcels from 4 items of the tenth factor, and another 2 parcels created from 4 items of the last/eleventh factor. It was assumed then that the reliabilities of the scores on the composites of two to three factors items forming one parcel would result to substantially greater reliabilities of scores.

**Table 1:** Descriptive statistics and internal consistency reliabilities of the Facilitating Conditions Questionnaire

FCQ Factors	Cronbach α		M		SD	
	English	Filipino	English	Filipino	English	Filipino
University intention (5 items)	0.88	0.83	4.46	4.55	0.67	0.55
School valuing (9 items)	0.87	0.82	4.40	4.40	0.58	0.51
Parent support (6 items)	0.88	0.83	3.25	3.79	0.96	0.81
Teacher support (6 items)	0.77	0.76	3.53	3.88	0.67	0.62
Peer help (5 items)	0.90	0.81	3.83	3.93	0.82	0.68
Leave school (4 items)	0.90	0.92	1.91	1.71	1.06	1.05
Pride from others (4 items)	0.84	0.87	3.87	3.83	0.83	0.90
Negative parent influence (5 items)	0.89	0.90	1.91	1.85	0.98	1.02
Affect to school (3 items)	0.77	0.57	3.67	4.01	0.81	0.76
Negative peer influence (4 items)	0.91	0.88	2.11	1.88	1.05	1.03
Positive peer influence (4 items)	0.84	0.82	4.18	4.33	0.75	0.71
Sense of Self Scale						
Sense of purpose (6 items)	0.85	0.78	4.37	4.53	0.58	0.49
Positive self-concept (5 items)	0.76	0.67	3.25	3.67	0.71	0.60
Negative self-concept (7 items)	0.79	0.78	2.68	2.58	0.73	0.72

In order to test the validity of the FCQ scale, a CFA was conducted with the following model: each of the eleven factors (university intention, school valuing, parent support, teacher support, peer help, leave school, pride from others, negative parent influence, affect to school, negative peer influence, and positive peer influence) of the FCQ served as the latent variables, and the manifest variables are the respective item parcels generated from the array of FCQ items linked to the latent variables. Likewise, the eleven scales (latent variables) were allowed to be freely correlated in the model. Separate CFAs were conducted for the English and translated Filipino versions of the FCQ. The Statistica 8 software was used for the entire statistical analysis. A number of goodness-of-fit indexes were used in this study. They include: chi-square (χ^2^), chi-square to degrees of freedom ratio (*χ* ^2^/df), root mean square error of approximation (RMSEA), non-normed fit index (NNFI), comparative fit index (CFI), goodness-of-fit index (GFI), and normed fit index (NFI).

Next, we tested for the equivalence of the two language versions using multigroup CFA. We followed a forward, stepwise approach, also called sequential constraint imposition (Dimitrov 2010). For our case, three levels of invariance were tested: configural invariance, measurement invariance, and structural invariance (Byrne 2010). First, we tested for configural invariance which tests whether the number of factors and pattern of indicator-factor loadings is identical across the Filipino and English versions. The configural invariance model is the model in which the same pattern of fixed (zero) and free factor loadings is specified for each of the two language versions and is considered the “minimal condition for factorial invariance” (Marsh 1993, p. 851). It provides the basis for comparison with all and provides the basis for comparison with all subsequent models. Second, we tested for measurement invariance where the factor loadings were constrained to be equal. This was done after we have established the configural invariance. Note that we only tested for invariant factor loadings in this stage and did not constrain the indicator intercepts to be equal because we were not interested in testing for differences in the latent means across the two versions. Third, we tested for structural invariance where equality constraints were placed on the factor variances and covariances across the two language versions. We did not test for equality of error variances and covariances across the two language versions because such a test is considered to be excessively stringent (Byrne 2010). The classical approach in arguing for evidence of invariance is based on *χ* ^2^ difference (Bollen 1989; Hu and Bentler 1995); however, from a more practical perspective Cheung and Rensvold (2002) claimed that it is more reasonable to base invariance decisions on a difference in CFI. In line with this, we followed Cheung and Rensvold’s (2002) criteria indicating that a decrease of 0.01 in the comparative fit index (CFI) is evidence for lack of invariance.

To test the between-network validity of the two language versions of the FCQ, we examined the relationships between the 11 FCQ factors and specific dimensions of the SOS Scale such as sense of purpose, positive and negative self-concept. It was hypothesized that the positive factors of the FCQ (e.g., parent support, teacher support, pride from others, etc.) would be associated with the positive aspects of the SOS Scale (sense of purpose and positive self concept); whereas the negative factors of the FCQ (e.g., negative parent influence, negative peer influence) would be associated with the negative factor of the SOS Scale (i.e., negative self concept). Moreover, factors of opposing valences should be negatively correlated with each other (e.g., negative peer influence would be negatively related to sense of purpose).

## Results

### Preliminary Analyses

Cronbach’s alpha coefficients were calculated for each of the scales of the FCQ. Reliability estimates were acceptable and varied from 0.84 to 0.91 for the English version. In general, the reliability estimates for the Filipino version were acceptable except for one factor *Affect to* *School* where the Cronbach’s alpha was 0.57 which is considered less than adequate. The rest of the dimensions in the Filipino version had reliability coefficients ranging from 0.77 to 0.92. The complete descriptive statistics for the two versions of the FCQ as well as the Sense of Self Scale are presented in Table 1.

### Within-Network Study Using CFA

*English version*. All of the fit indexes were adequate: RMSEA = 0.03, NNFI =. 988, CFI = 0.992, GFI = 0.975, NFI = 0.976. These values show good fit (Byrne 2010). Of all the goodness-of-fit indicators considered in this study, only the chi-square (*χ* ^2^ (38) = 56.086, *p* < 0.001; *χ* ^2^/df = 1.48) was not adequate. A significant *χ* ^2^ value indicates bad fit. However, as discussed by Anderson and Gerbing (1988), and Huang and Michael (2000), the value of the chi-square is directly dependent on sample size. Because of this, with a large sample size, significant values can be obtained even though there are only trivial discrepancies between the model and the data. Thus, we decided to focus on the other fit indices which all indicated a good fit.

*Filipino version*. The results of the CFA on the Filipino version of the FCQ also indicated good fit: RMSEA = 0.02; NNFI = 0.983, CFI = 0.991, GFI = 0.965, NFI = 0.971.

Bivariate correlations among the different factors of the FCQ were also obtained for both the English and Filipino versions (see Tables 2 and 3).

**Table 2:** Zero-order correlations among latent variables of the Facilitating Conditions Questionnaire — English version

FCQ factors	2	3	4	5	6	7	8	9	10	11
1. University Intention	0.63^***^	0.00	0.17^**^	0.29^***^	−0.32^***^	0.25^***^	−0.25^***^	0.19^***^	−0.25^***^	0.45^***^
2. School Valuing	–	0.070	0.33^***^	0.27^***^	−0.28^***^	0.42^***^	−0.32^***^	0.39^***^	−0.26^***^	0.47^***^
3. Parent Support		–	0.24^***^	0.17^**^	−0.17^**^	0.17^**^	0.03	0.26^***^	0.06	0.00
4. Teacher Support			–	0.42^***^	−0.13^*^	0.38^***^	0.01	0.37^***^	0.10^*^	0.15^**^
5. Peer Help				–	−0.06	0.34^***^	−0.09	0.13^*^	−0.01	0.23^***^
6. Leave School					–	−0.11^*^	0.66^***^	−0.02	0.61^***^	−0.31^***^
7. Pride From Others						–	−0.23^***^	0.25^***^	−0.08	0.33^***^
8. Negative Parent Influence							–	−0.10	0.55^***^	−0.32^***^
9. Affect to School								–	0.03	0.25^***^
10. Negative Peer Influence									–	−0.24^**^
11. Positive Peer Influence										–

*p < 0.05; ** *p* < 0.01; *** *p* < 0.001

**Table 3:** Zero-order correlations among latent variables of the Facilitating Conditions Questionnaire — Filipino version

FCQ factors	2	3	4	5	6	7	8	9	10	11
1. University Intention	0.54^***^	0.11^*^	0.39^***^	0.26^***^	−0.25^***^	0.28^***^	−0.32^***^	0.20^***^	−0.26^***^	0.36^***^
2. School Valuing	–	0.24^***^	0.45^***^	0.37^***^	−0.23^***^	0.40^***^	−0.38^***^	0.36^***^	−0.27^***^	0.37^***^
3. Parent Support		–	0.33^***^	0.26^***^	0.07	0.31^***^	−0.01	0.22^***^	0.11^*^	0.08
4. Teacher Support			–	0.57^***^	−0.02	0.46^***^	−0.03	0.44^***^	−0.02	0.30^***^
5. Peer Help				–	−0.05	0.35^***^	−0.06	0.34^***^	−0.07	0.33^***^
6. Leave School					–	−0.09	0.69^***^	−0.15^**^	0.65^***^	−0.22^***^
7. Pride From Others						–	−0.16^**^	0.32^***^	0.02	0.28^***^
8. Negative Parent Influence							–	−0.15^**^	0.66^***^	−0.32^***^
9. Affect to School								–	−0.09	0.31^***^
10. Negative Peer Influence									–	−0.29^***^
11. Positive Peer Influence										

*p < 0.05; ** *p* < 0.01; *** *p* < 0.001

A look at the correlations among the latent factors in the FCQ revealed slight variations. Generally it can be said that there were slight differences in the correlation patterns among the latent variables of the data in both the language versions. While there were latent variables which were significantly correlated with other latent variables in the two language versions, there were also some variables which either registered significant correlation in one language version but not in the other language version and vice versa.

### Multigroup Confirmatory Factor Analysis

The first multigroup CFA for the English and Filipino versions allowed all factor loadings, uniquenesses, and correlations to be freely estimated. This model yielded a good fit to the data (see Table 4). After finding support for configural invariance, we tested for measurement invariance by holding the factor loadings invariant across the two language versions. Results in Table 4 indicate that the factor loadings for the two measures were invariant, because the drop in CFI was <0.01. We then tested for a third model where we held both factor loadings and factor variances and covariances invariant across the two groups; however, we found evidence of non-invariance. The drop in CFI was greater than 0.01; however, the fit indices still seem to be acceptable (i.e., the TLI and CFI are well above 0.90 and RMSEA is below 0.05).

**Table 4:** Goodness-of-fit indices for the Multigroup Analysis

Model	*χ* ^2^	*df*	*χ* ^2^/*df*	*p*	RMSEA	NNFI/TLI	CFI	Change in CFI
1. Baseline model (no invariance imposed)	608.50	350	1.739	*p* < 0.001	0.031	0.970	0.980	–
2. Invariant factor loadings	674.30	362	1.863	*p* < 0.001	0.034	0.965	0.975	0.005
3. Invariant factor variances and covariances	928.75	428	2.170	*p* < 0.001	0.039	0.953	0.960	0.015

*RMSEA* root mean square error of approximation; *NNFI/TLI* non-normed fit index or Tucker-Lewis Index; *CFI* comparative fit index

### Between-Network Construct Validation

The results of the between-network analysis generally revealed the same pattern of results for the English and Filipino versions (see Table 5). The positive dimensions of the FCQ were generally positively related with the positive aspects of the SOS Scale (positive self-concept and sense of purpose), and some were also negatively correlated with the negative factor of the SOS Scale (negative self-concept). On the other hand, the negative dimensions of the FCQ (negative parent influence and negative peer influence) were likewise positively correlated with negative self-concept, and negatively correlated with sense of purpose (but not with positive self-concept).

**Table 5:** Zero-order correlations of the FCQ with sense of self dimensions

FCQ subscale	Sense of purpose		Negative self concept		Positive self concept	
	English	Filipino	English	Filipino	English	Filipino
1. University Intention	0.51^***^	45^***^	−0.22^***^	−0.19^***^	0.30^***^	0.26^***^
2. School Valuing	0.59^**^	0.54^***^	−0.12^*^	−0.23^***^	0.23^***^	0.37^***^
3. Parent Support	0.07	0.11^*^	0.01	0.08	0.06	0.22^***^
4. Teacher Support	0.29^***^	0.31^**^	−0.02	−0.05	0.14^***^	0.29^***^
5. Peer Help	0.20^***^	0.28^***^	0.12^*^	−0.06	0.05	0.21^***^
6. Leave School	−0.24^***^	−0.21^***^	0.26^***^	0.24^***^	−0.05	−0.01
7. Pride from others	0.28^***^	0.29^***^	−0.07	−0.11^*^	0.19^***^	0.35^***^
8. Negative Parent Influence	−0.21^***^	−0.29^***^	0.32^***^	0.34^***^	−0.04	−0.12^*^
9. Affect to School	0.35^***^	39^***^	−0.06	−0.09	0.23^***^	0.29^***^
10. Negative Peer Influence	−0.19^***^	−0.18^***^	0.31^***^	0.32^***^	0.07	−0.00
11. Positive Peer Influence	0.25^***^	0.31^***^	−0.15^**^	−0.17^***^	0.21^***^	0.23^***^

*p < 0.05; ** *p* < 0.01; *** *p* < 0.001

## Discussion

This study was conducted to validate the original English and the new Filipino translation of the 55–item Facilitating Conditions Questionnaire (FCQ) using a large sample of Filipino adolescents studying in high schools in the Philippines. The results provide good evidence for the configural validity of the eleven-factor structure of the ISM in both language versions, and sufficient evidence for invariance of the two language versions. The results also provide preliminary evidence for the construct validity of the two language versions by examining the nomological network of the FCQ scales as they relate to the students’ sense of self in the school context.

In general, results of both the between-network and within-network studies showed that both language versions were applicable for the bilingual Filipino adolescents in the study. In terms of the between-network studies, the CFAs showed good fit indices. The CFA approach used in this study provided a stronger validation compared to previous research on non-Western samples which just used Cronbach’s alpha reliability as a measure of internal consistency (e.g., McInerney 2008). Results of the multigroup analysis revealed configural invariance and also invariance of the factor loadings. However, the correlations among the factors were shown to be not invariant. This is possibly due to the slight differences in the correlations among the factors in the English and Filipino versions. Future research is needed to determine whether these slight differences are substantive or just results of sampling idiosyncracies. In terms of between-network validity, we found that the pattern of correlations between the FCQ constructs and the sense of self scales were generally similar.

However, there were also some interesting differences between the two language versions. First is the big difference in the internal consistency of the Affect to School subscale in the two language versions. It should be noted that *affect to* *school* for the English version had a Cronbach’s alpha of 0.77 and 0.57 for the Filipino version. Reflecting on this result, there are several defensible ways in which this finding could be interpreted. First, on the part of the Filipino adolescents, it seems clear that the items of the English version in the mentioned construct were more clearly understood by them in contrast with the items in the Filipino version. Perhaps this possibility can be expected considering the educational experiences of Filipino. English is the medium of instruction in the Philippines and only few subjects are taught in Filipino. In keeping with this practice, many schools have strictly implemented a speak-in-English policy within the boundaries of the schools and the possibility of producing students who have better facility for English language rather than Filipino language is possible. Another possibility is that the Filipino items were not constructed properly and that they may need to be revised to be more suited to the language and comprehension level of the Filipino adolescents.

The FCQ can be a useful instrument for future research with school-based adolescents in the Philippines. Researchers who are interested in measuring the social support received by the adolescent students from their significant others could use the FCQ in their studies. This is relevant because most of the available measurement tools in the literature measure only internally-referenced motivational constructs. Examples include the well-cited Motivated Strategies for Learning Questionnaire (Pintrich and DeGroot 1990), the Patterns of Adaptive Learning Survey (Midgley et al. 2000), the Achievement Goal Questionnaire (Elliot and McGregor 2001), and the Self-Description Questionnaire (Marsh 1988) among others. However, there are fewer instruments that measure externally-referenced constructs such as those that relate to peers, teachers, and parents. Thus the cross-cultural validation of the FCQ which can appropriately measure both internally and externally-referenced motivational constructs is especially helpful.

### Limitations and Directions for Future Research

We should note that there are some limitations of the study. Ideally the cross-linguistic validity of the translations could be assessed using a bilingual sample that answers both language versions. However, in our study, a different set of students answered each language version. Despite this, we had no reason to suspect that there were marked differences between the two groups as students were randomly assigned to the English or Filipino group. Another limitation is that we only tested the relationship of the FCQ to sense-of-self variables for the between-network validity. Future studies can explore the relationship of the FCQ to other relevant variables such as academic achievement among others. In our study, we only validated the FCQ among high school Filipino students, thus we cannot make any claims about the applicability of the two language versions among Filipino children in elementary schools. Future studies could test the FCQ among children in upper elementary. In this study, the FCQ was tested among students from the general high school population. However, future research could also consider using the FCQ with high-risk students. As Anderson and Keith (1997) noted, “…student motivation may have a stronger impact on at-risk students’ achievement than on the achievement of high school students in general” (p. 259). It is possible that motivation may even be a more salient issue for high-risk students who live in impoverished and stressful environments (Becker and Luthar 2002; Gordon-Rouse 2002; Whitaker et al. 2012).

In terms of the statistical analyses, we used methods associated with classical test theory (i.e. CFA) to test for the validity of the instrument. However, other possible approaches such as those using Item Response Theory (IRT) can also be used. Future studies could also use IRT techniques in order to test for possible differential item functioning (DIF) in different versions of the questionnaire.

## Conclusions

These limitations notwithstanding, the results of the study provide further evidence supporting the validity of the FCQ across cultures, and in this case across two language versions in a bilingual culture. Note that although this is not the first study that validated the FCQ in another culture, this is the first study that tested the invariance the FCQ in two language versions among a bilingual population of adolescent students. Thus, this study contributes to further validation of the FCQ as a psychological tool that can be used in a variety of educational contexts and cultures. Our results indicate that there is strong support for the assumption that the FCQ measures the same externally-referenced motivational forces in both language versions, and that the relationship between these constructs and other external constructs in the nomological network are comparable in both versions.

It would be interesting for future studies to use the FCQ to measure the social support accorded to Filipino students of varying cultural groups such as those from the majority group and ethnic minority groups. McInerney (2008) has previously used the FCQ among different minority groups in Australia and the U.S. to assess which factors are responsible for the lower academic achievement of majority versus minority groups. In the Philippines, participation rates are lower in regions with high proportions of ethnic minority groups (e.g., indigenous ethnic groups in the Cordillera region and ethnic Muslim groups in Mindanao, Caoli-Rodriguez 2007). Researchers may explore the similarities and differences of facilitating conditions vis-a-vis achievement outcomes and whether there were differences between cultural groups on the salience of the perceived facilitating conditions.

In a recent discussion of the cross-cultural applicability of motivation theories across different contexts, Zusho and Clayton (2011) noted the need to conduct research on a wider range of cultures. We think that our effort to validate two language versions of the FCQ for use with Filipino students is a useful step in this direction.

## Appendix

### Facilitating Conditions Questionnaire-English and Filipino Versions

**Table:**

	English version	Filipino Version
University intention	1. I intend to go on to college or university	1. Intensiyon kong ipagpatuloy ang pag-aaral sa kolehiyo o unibersidad.
University intention	2. Most people who are important to me think that I should go to college or university	2. Karamihan sa mga taong importante sa akin iniisip na dapat akong magpatuloy sa kolehiyo o unibersidad.
University intention	3. I’m the kind of person who would go to college or university	3. Ako ang klase ng tao na magpapatuloy sa kolehiyo o unibersidad.
University intention	4. I’m the kind of person who would complete college or university	4. Ako ang klase ng tao na magtatapos ng kolehiyo o unibersidad.
University intention	5. I personally feel that I should complete college or university	5. Personal kong nararamdaman na dapat akong magtapos sa kolehiyo o unibersidad.
School valuing	6. Education is important for me to get a job	6. Ang edukasyon ay importante sa akin para makakuha ng trabaho.
School valuing	7. People who have a good schooling get more out of life than ones who don’t	7. Ang mga taong may magandang pinag-aralan ay may mas mararating sa buhay kaysa sa mga taong hindi.
School valuing	8. If I do well at school I am more likely to get a good job	8. Kung pinaghuhusay ko ang aking pag-aaral, mas malamang na ako ay makakakuha ng magandang trabaho.
School valuing	9. I think that it is really important to do well at school	9. Iniisip ko na talagang importante na maging mahusay sa paaralan.
School valuing	10. Doing well at school is really important to my future	10. Ang pagiging mahusay sa paaralan ay importante sa aking kinabukasan.
School valuing	11. It’s important for me to do well at school	11. Importante sa akin na maging mahusay sa paaralan.
School valuing	12. School students should complete high school	12. Ang mga mag-aaral ay dapat matapos sa hayskul.
School valuing	13. Many of the subjects I learn at school will help me after I leave school	13. Marami sa mga “subjects” na natutunan ko sa paaralan ay makatutulong sa akin kapag ako ay nakapagtapos na sa pag-aaral.
School valuing	14. What I learn at school will be useful after I leave school	14. Ang anumang aking natutunan sa paaralan ay makatutulong kapag ako ay nakapagtapos na sa pag-aaral.
Parent support	15. My mother helps me with my schoolwork	15. Tinutulungan ako ng aking ina sa aking “schoolwork”.
Parent support	16. My father helps me with my schoolwork	16. Tinutulungan ako ng aking ama sa aking “schoolwork”.
Parent support	17. It’s important to me to have my mother’s help with schoolwork	17. Importante sa akin ang pagtulong ng aking ina sa aking “schoolwork”.
Parent support	18. It’s important to me to have my father’s help with schoolwork	18. Importante sa akin ang pagtulong ng aking ama sa aking “schoolwork”.
Parent support	19. My father helps me to work hard at school	19. Tinutulungan ako ng aking ama na magsumikap sa paaralan.
Parent support	20. My mother helps me to work hard at school	20. Tinutulungan ako ng aking ina na magsumikap sa paaralan.
Teacher support	21. Teachers are positive to me at school	21. Ang mga guro ko ay positibo sa akin sa paaralan.
Teacher support	22. I get encouragement from some of my teachers to do well at school	22. Nakakakuha ako ng “encouragement” sa ilan sa aking mga guro na maging mahusay sa paaralan.
Teacher support	23. If I decided to go on to college or university teachers at this school would encourage me	23. Kapag ako ay nagpasya na magpatuloy sa kolehiyo o unibersidad, ang mga guro sa paralang ito ay “mag-eencourage” sa akin.
Teacher support	24. My teachers help me with my schoolwork	24. Tinutulungan ako ng aking guro sa aking “schoolwork”.
Teacher support	25. It’s important to me to have my teacher’s help with schoolwork	25. Importante sa akin ang mabigyan ng tulong ng aking mga guro sa aking mga “schoolwork”.
Teacher support	26. My teachers help me to work hard at school	26. Tinutulungan ako ng aking mga guro na magsumikap sa paralan.
Peer help	27. Some of my friends help me with my schoolwork	27. Ang ilan sa aking mga kaibigan ay tinutulungan ako sa aking “schoolwork”.
Peer help	28. My friends help me with my schoolwork	28. Tinutulungan ako ng aking mga kaibigan sa aking mga “schoolwork”.
Peer help	29. It’s important to me to have my friend’s help with schoolwork	29. Importante sa akin na mabigyan ng tulong ng aking mga kaibigan sa aking “schoolwork”.
Peer help	30. Working with my friends at school improves my schoolwork	30. Ang pagtatrabaho kasama ng aking mga kaibigan ay nagpapabuti sa aking “schoolwork”.
Peer help	31. My friends help me to work hard at school	31. Tinutulungan ako ng aking mga kaibigan na magsumikap sa paaralan.
Leave school	32. My mother doesn’t mind if I leave school when I want to	32. Balewala sa aking ina kung ako’y hihinto sa paaralan kung kailan ko gusto
Leave school	33. My father doesn’t mind if I leave school when I want to	33. Balewala sa aking ama kung ako’y hihinto sa paaralan kung kailan ko gusto
Leave school	34. My father thinks that I should leave school and get a job	34. Iniisip ng aking ama na dapat akong tumigil sa pag-aaral at magtrabaho.
Leave school	35. My mother thinks that I should leave school and get a job	35. Iniisip ng aking ina na dapat akong tumigil sa pag-aaral at magtrabaho.
Pride from others	36. It’s important for my father to be proud of my schoolwork	36. Importante sa aking ama na maipagmalaki ang aking “schoolwork”.
Pride from others	37. It’s important for my mother to be proud of my schoolwork	37. Importante sa aking ina na maipagmalaki ang aking “schoolwork”.
Pride from others	38. It’s important for my teachers to be proud of my schoolwork	38. Importante sa aking guro ang maipagmalaki ang aking “schoolwork”.
Pride from others	39. It’s important for my friends to be proud of my schoolwork	39. Importante sa aking mga kaibigan ang maipagmalaki ang aking “schoolwork”.
Negative parent influence	40. My father doesn’t pay any attention when I bring home report cards	40. Hindi binibigyang pansin ng aking ama kapag inuuwi ko sa bahay ang aking “report card”.
Negative parent influence	41. My mother doesn’t pay any attention when I bring home report cards	41. Hindi binibigyang pansin ng aking ina kapag inuuwi ko sa bahay ang aking “report card”.
Negative parent influence	42. My mother doesn’t care if I get a job or not when I leave school	42. Walang pakialam ang aking ina kung magkaroon man ako o hindi ng trabaho pagkatapos ng pag-aaral.
Negative parent influence	43. My father doesn’t care if I get a job or not when I leave school	43. Walang pakialam ang aking ama kung magkaroon man ako o hindi ng trabaho pagkatapos ng pag-aaral.
Negative parent influence	44. I don’t care if I get a job or not when I leave school	44. Wala akong pakialam kung magkaroon man ako ng trabaho o hindi kapag ako’y huminto sa pag-aaral.
Affect to school	45. I like studying	45. Gusto kong nag-aaral.
Affect to school	46. I like working at school	46. Gusto kong nagtatrabaho sa paaralan.
Affect to school	47. Subjects at school interest me	47. Ang mga subjects sa paaralan ay nagbibigay “interest” sa akin.
Negative peer influence	48. Some of my friends tell me I should leave school when I can	48. Ilan sa mga kaibigan ko ang nagsasabing dapat na akong tumigil sa pag-aaral kung kaya ko.
Negative peer influence	49. Some of my friends tell me to leave school and to get a job	49. Ilan sa aking mga kaibigan ang nagsasabing tumigil na ako sa pag-aaral at magtrabaho na lang.
Negative peer influence	50. Some of my friends leave school early and go on welfare	50. Ilan sa aking mga kaibigan ay maagang tumigil sa pag-aaral at umasa na lamang sa iba.
Negative peer influence	51. Some of my friends want to leave school as soon as they can	51. Ilan sa mga kaibigan ko ay gusto ng tumigil sa pag-aaral sa lalong madaling panahon.
Positive peer influence	52. Most students in my class will go on to college or university	52. Karamihan sa mga estudyante sa aming klase ay magpapatuloy sa kolehiyo o universidad.
Positive peer influence	53. Most of my friends want to do well at school	53. Karamihan sa aking mga kaibigan ay gustong maging mahusay sa paaralan.
Positive peer influence	54. Some of my friends want to go on to college or university	54. Ang ilan sa aking mga kaibigan ay gustong magpatuloy sa kolehiyo o unibersidad.
Positive peer influence	55. Most students in my class will complete high school	55. Karamihan sa mga estudyante sa aming klase ay magtatapos sa hayskul.
